# High prevalence of non-alcoholic fatty liver disease in patients with a first episode of acute ischemic stroke. Impact on disability and death

**DOI:** 10.3389/fendo.2022.1003878

**Published:** 2022-12-16

**Authors:** Lidia Canillas, Agnes Soriano-Varela, Ana Rodríguez-Campello, Eva Giralt-Steinhauer, Elisa Cuadrado-Godia, Teresa Broquetas

**Affiliations:** ^1^ Liver Section, Gastroenterology Department, Hospital del Mar, Institut Hospital del Mar d’Investigacions Mèdiques (IMIM), Barcelona, Spain; ^2^ Department of Medicine and Life Sciences (MELIS), Universitat Pompeu Fabra (UPF), Barcelona, Spain; ^3^ Stroke Unit. Neurology Department, Hospital del Mar, Institut Hospital del Mar d’Investigacions Mèdiques, Barcelona, Spain

**Keywords:** non-alcoholic fatty liver disease (NAFLD), metabolic-associated fatty liver disease (MAFLD), fatty liver index (FLI), acute ischemic stroke, prognosis, cardiovascular event

## Abstract

**Introduction:**

Non-alcoholic fatty liver disease (NAFLD) is the most common chronic liver disease, and it is associated with an increased risk of overall mortality being cardiovascular disease the most common cause of mortality. Strategies are needed to identify high risk groups for NAFLD to improve screening approaches. Moreover, there is a lack of information about the prevalence of NAFLD on patients with acute ischemic stroke (AIS) and the influence of NAFLD on the prognosis of the stroke. The aim of the study was to define the prevalence of NAFLD in patients with a first episode of AIS and the secondary aims were to evaluate the prevalence of NAFLD at different ages and its impact on the severity and prognosis of the AIS.

**Materials and methods:**

Observational study including consecutive patients admitted for the first AIS from January 2005 to May 2018. Patients with harmful alcohol intake, other liver diseases and malignancies were excluded. Sociodemographic data, cardiovascular risk factors, comorbidities, and blood test at admission were reviewed. NAFLD and liver fibrosis were assessed with the serological scores Fatty Liver Index (FLI) and Fibrosis-4 respectively. NAFLD was defined by a FLI>60. Stroke severity and prognosis were evaluated with the National Institute of Health Stroke Scale and modified Rankin Scale respectively in patients aged from 40 to 79 years old.

**Results:**

We included 1601 patients, 52.4% were female and median (IQR) age of 77 (66 – 83) years. The 41% of the total cohort had a FLI>60 with different prevalence according to age in decades: in 30-39 years: 35.7%; in 40-49: 47.5%; in 50-59: 51.1%, in 60-69: 56%, in 70-79: 41.4%; in 80-89: 34.9% (p<0.001). The presence of NAFLD did not impact on the severity or the prognosis of stroke. However, patients with NAFLD were younger than those without NAFLD (74 vs. 78; p<0.001).

**Conclusion:**

Presence of NAFLD did not impact on disability and death after the stroke. However, patients with a first episode of stroke showed a high prevalence of NAFLD, especially at intermediate ages, and therefore, screening for NAFLD should be advisable.

## Introduction

Non-Alcoholic Fatty Liver Disease (NAFLD) is the most common cause of chronic liver disease, with an estimated prevalence of 25% in the general population (1–3). It is strongly related to obesity and type 2 diabetes mellitus (T2DM), occurring in 50-90% of these patients (4, 5). Its incidence is expected to increase in the coming years due to the increasing prevalence of obesity and T2DM. Classically, NAFLD is defined by the accumulation of fat in more than 5% of hepatocytes in individuals without significant alcohol consumption or other liver diseases. The progression of the disease is determined by necroinflammation (steatohepatitis) and liver fibrosis, which is estimated to occur in 12-40% of patients, and which can eventually lead to the presence of cirrhosis and hepatocellular carcinoma (4, 6). Liver biopsy is the gold standard for the diagnosis of NAFLD and the stage of the disease, but because of its invasiveness the diagnosis is usually based on risk factors, laboratory tests, radiologic signs and transient elastography (1, 7). Fatty liver index (FLI) is a simple score based on body mass index (BMI), triglycerides, gamma-glutamyltransferase (GGT) and waist circumference. A FLI value >60 has a specificity of 86% to rule in NAFLD, while a value <30 a sensitivity of 87% to rule out NAFLD (8). Liver fibrosis is the most important prognostic factor in NAFLD and correlates with liver outcomes and mortality (9). Indeed, non-invasive serological and elastographic methods to evaluate liver fibrosis in NAFLD have been evaluated and validated, and they have been especially useful for excluding advanced fibrosis (1).

Patients with NAFLD have an increased risk of extrahepatic morbidity and mortality. The most common causes of death in patients with NAFLD are cardiovascular diseases (~40%), extrahepatic neoplasms (~20%), and liver complications (~10%) (2, 10, 11). Studies have shown that NAFLD is an independent risk factor for cardiovascular disease after adjusting for traditional risk factors (10, 12, 13), and, indeed, it seems that NAFLD and T2DM could act synergistically in triggering cardiovascular events (14). Besides, while some literature suggested that NAFLD patients with liver fibrosis (evaluated with serologic markers or histology) were reported to have between 2 and 4-fold increased risk of incident cardiovascular events (13, 15), other authors could not demonstrate this association assessing liver fibrosis by elastography (16).

Stroke is the second leading cause of death and disability-adjusted life years (17). The prevalence of NAFLD in patients suffering from acute ischemic stroke (AIS) ranged from 18-36% in prospective studies depending on different diagnostic tools used (serological, image techniques and histology) (18, 19). Different meta-analysis of observational cohorts and case-control studies reported higher overall risk of stroke in NAFLD patients, with an estimated risk of 1.1 to 2.5 times the risk of patients without NAFLD (20–23). However, there is limited evidence of the role of NAFLD in the severity and prognosis of AIS. On the other hand, it has been described that age is a prognostic factor in stroke outcome. Older patients have a poorer stroke outcome with higher fatality rate and longer length of hospital stay (24).

Hence, the primary aim of our study was to assess the prevalence of NAFLD in a cohort of European patients with a first AIS. The secondary aims were to evaluate the prevalence of NAFLD at different ages and the relationship between NAFLD and the severity and prognosis of the AIS in patients aged between 40 and 79 years old.

## Materials and methods

### Study population

This is an observational study including consecutive patients admitted to our tertiary centre (Hospital del Mar, Barcelona, Spain) for a first episode of AIS from January 2005 to May 2018. Exclusion criteria were alcohol consumption above 30g/day in men and 20g/day in women (25), another chronic liver disease, an active malignancy (26), intracranial hemorrhagic events and a previous transient ischemic attack. All patients provided written informed consent, or it was obtained from their designated representative to be included in a prospective ongoing registry (BasicMar), that includes all the patients admitted for episode of stroke in our institution. The study protocol was approved by the Ethical Committee of our institution “Comitè Ètic d’Investigació Clínica - Parc de Salut Mar”, study reference (2008/3083/I) in accordance with the ethical guidelines of the 1975 Declaration of Helsinki.

### Stroke definition, etiology, severity and prognostic variables

Neurologic examination was performed in the Emergency Care Unit. The stroke diagnosis was made by a Neurologist according to the World Health Organization criteria (27). All patients were evaluated at hospital admission by a vascular-trained neurologist to establish initial severity. During the admission, additional examinations were carried out to define the etiology of the AIS. Stroke etiology was classified as large artery disease, cardioembolic, small vessel disease, undetermined, infrequent or unclassified according to the causative classification system (28). Ischemic strokes of infrequent cause and unclassified have been grouped in the others category by their low frequency.

Stroke severity was defined by the National Institute of Health Stroke Scale (NIHSS), which was evaluated at admission and at discharge. NIHSS assesses the impairment after a stroke by a numerical 15-item scale. Each item is scored on an ordinal scale and scores are summed to a total score ranging from 0 to 42. A higher score reflects a more severe stroke. Based on previous studies, severity was considered as mild if NIHSS<7, moderate if NIHSS ranges between 7-14 and severe if NIHSS was >14 (29).

Prognosis was evaluated at 90 days of the stroke, either in-person or *via* telephone interview, with the patient or relatives by the modified Rankin Scale (mRS), that measures from 0 to 6 the functional status and disability after stroke (30, 31). mRS values of 0-2 are considered as no or slight disability, mRS 3-5 means moderate to severe disability and mRS 6 means death (32). Poor outcome was defined as disability or death (mRS 3-6).

### Clinical variables

Sociodemographic data, smoking habit, pre-existing disability, and comorbidities [hypertension, T2DM, dyslipidemia, BMI and atrial fibrillation (AF)] were recorded. Alcohol intake data was obtained directly from the patient (or from relatives in cases where this was not possible) and, when possible, was compared with data recorded in electronic medical records by the patient’s general practitioner or obtained by other physicians in our center to certify the consistency of information. Anthropometric measurements were collected during admission. Blood tests were performed in the emergency room or during admission.

### NAFLD and liver fibrosis evaluation

NAFLD presence was assessed by FLI. The FLI was calculated using the formula published by Bedogni et al. It ranges from 0 to 100. A FLI >60 was used to rule in NAFLD (8). Liver fibrosis was assessed by Fibrosis-4 (FIB-4), that was calculated according to the formula previously published (33). To rule out advanced fibrosis, the cut-offs used were adjusted to age (FIB-4 <1, 3 and FIB-4 <2 in those aged < 65 years and ≥65 years respectively). FIB-4>2.67 cut-off was used to rule-in advanced fibrosis (34). The evaluation of the impact of NAFLD and fibrosis scores on the severity and prognosis of stroke was focused on patients aged from 40 to 79 years. The decision to focus this evaluation on patients at intermediate ages was because two main concerns. On one hand, as described above, age is a well-recognized independent risk factor for poor prognosis of stroke (24) and on the other hand, due to the risk of infra and overestimation of liver fibrosis evaluated by FIB-4, at extreme ages (34).

### Statistical analysis

In the descriptive analysis, quantitative variables were expressed as medians and interquartile range (IQR), and categorical variables as absolute number (n) and proportions (%). Variables were compared between groups using Chi-square, U Mann-Whitney and Kruskal-Wallis tests as appropriate. Multivariate analysis was performed using binary logistic regression to test severity and prognosis. Covariates that were significant with p<0.05 in univariate analysis were included in multivariate model. We performed two models of multivariate analyses, one including the composite scores (i.e.FIB-4) and the other including the variables for FIB-4 calculation entered individually to avoid collinearity. All the analyses were 2-tailed and a p-value <0.05 was considered statistically significant. Statistical analysis and graphs were performed using STATA V14.2 (StataCorp).

## Results

### Baseline characteristics

**Figure 1:**
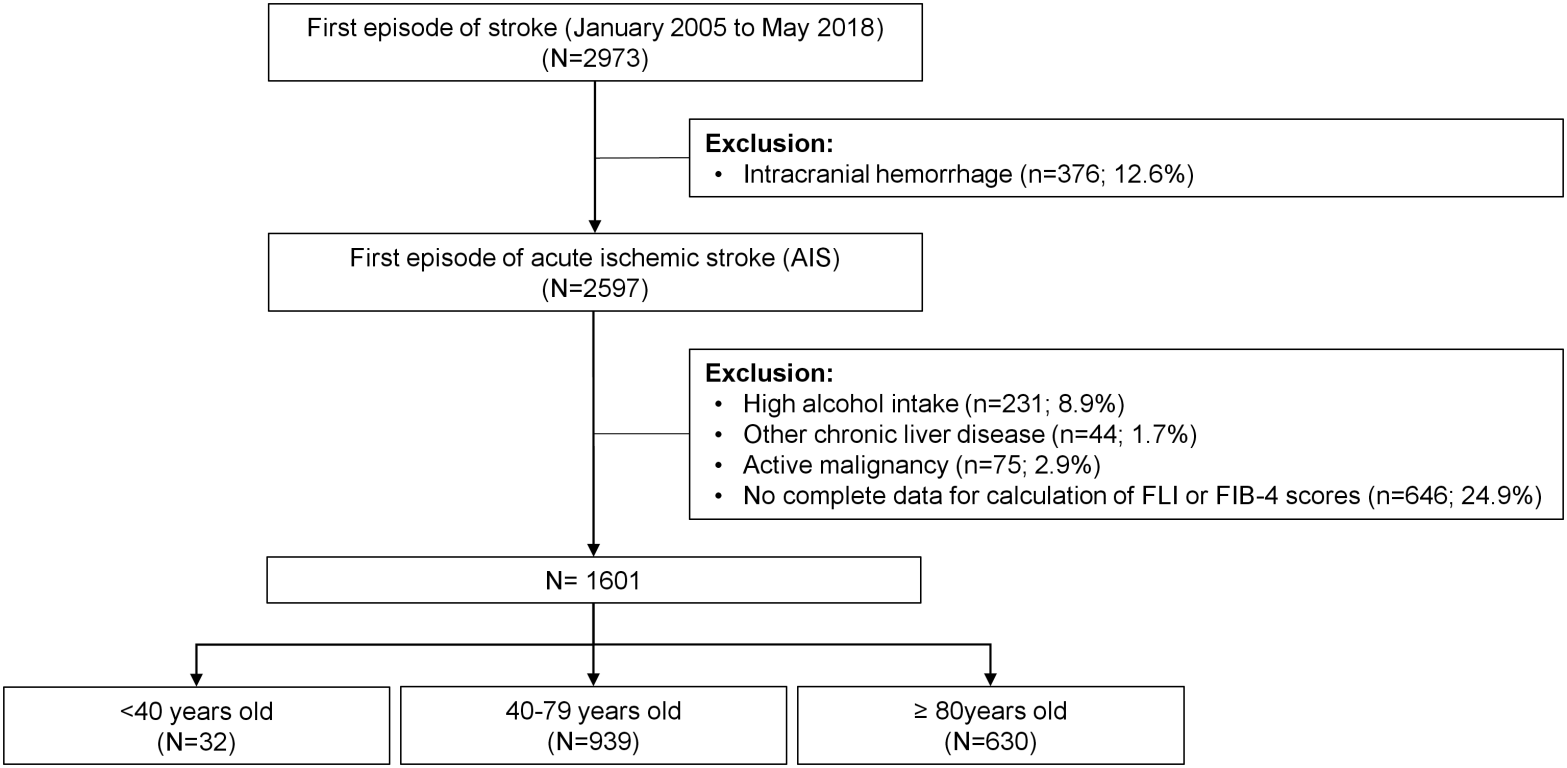
Flow-chart of patients included in the study.

From January 2005 to May 2018, 2973 patients were admitted to our hospital for a first episode of stroke. Patients with intracranial hemorrhage (n=376), high alcohol intake (n=231), other chronic liver disease (n=44) and an active malignancy (n=75) were excluded. In 646 patients, there were no complete data to perform FLI or FIB-4 scores, or the mRS was not registered at follow-up. Therefore, 1601 patients were included in the analyses. **Figure 1** shows the flow chart of patients.

Main characteristics of included patients are depicted in **Table 1**. Median (IQR) age was 77 (66 – 83) years and 52.4% of patients were female. Cardioembolic etiology was the predominant cause of the stroke, present in the 35.3% of patients. Median BMI was 26.8kg/m^2^, and 421 (26.3%), 535 (33.4%) and 1211 (75.6%) patients had obesity, T2DM and hypertension, respectively. Five hundred seventy-seven patients (36%) had AF. **Table 2** details the prevalence of stroke risk factors according to age at the stroke. Hypertension and specially AF were highly prevalent in elderly patients (>80 years old), while in younger patients the prevalence of T2DM, obesity and dyslipidemia were more frequent.

**Table 1:** Basal characteristics of the 1601 patients included in the analysis. Continuous variables are expressed with median (percentile 25 – percentile 75), and categorical variables as count (percentage).

	N = 1601
SOCIODEMOGRAPHIC DATA AND COMORBIDITIES	
** Age** (years)	77 (66 – 83)
** Female**, n (%)	839 (52.4)
** Caucasian ethnicity**, n(%) (n=1575)	1491 (94.7)
** Tobacco habit**, n (%) (n=1576	
** **Never smoker	1043 (66.2)
** **Ex-smoker > 5 years	198 (12.6)
** **Active smoker or ex-smoker < 5 years	335 (21.3)
** Abdominal circumference** (cm)	98 (90 – 108)
** BMI** (kg/m2)	26.8 (24.2 – 30.2)
** **BMI <25 kg/m^2^, n (%)	535 (33.4)
** **BMI 25 - 30 kg/m^2^, n (%)	645 (40.3)
** **BMI >30kg/m^2^, n (%)	421 (26.3)
** Hypertension**, n (%)	1211 (75.6)
** Type 2 Diabetes**, n (%)	535 (33.4)
** Dyslipidemia**, n (%)	799 (49.9)
** Atrial fibrillation**, n (%)	577 (36.0)
BLOOD TEST	
** AST** (U/L)	18 (15 – 24)
** ALT** (U/L)	15 (12 – 21)
** GGT** (U/L)	23 (15 – 38)
** Albumin** (g/dl) (n=1217)	3.8 (3.5 – 4.0)
** Platelets** (·10^9^/L)	219.0 (183.0 – 265.0)
** HbA1c** (%) (n=1347)	5.8 (5.3 – 6.6)
** Total cholesterol** (mg/dl) (n=1562)	172 (142 – 200)
** Triglycerides** (mg/dl)	111 (84 – 147)
STROKE-RELATED VARIABLES	
** Stroke ethiology**, n (%)	
** **Large artery disease	190 (11.9)
** **Cardioembolic	565 (35.3)
** **Small vessel disease	355 (22.2)
** **Undetermined	299 (18.7)
** **Others	192 (12.0)
** NIHSS**, n (%) (n=1592)	
** **<7	1023 (64.3)
** **7 – 14	309 (19.4)
** **>14	260 (16.3)
** Rankin**, n (%)	
** **0 – 2	912 (57.0)
** **3 – 5	521 (32.5)
** **6	168 (10.5)
LIVER-RELATED VARIABLES	
** FLI**	51.4 (27.5 – 75.7)
** **FLI <30, n (%)	443 (27.7)
** **FLI 30 – 60, n (%)	500 (31.2)
** **FLI ≥60, n (%)	658 (41.1)

BMI, body mass index; AST, aspartate aminotransferase; ALT, alanine aminotransferase; GGT, gamma glutamyl transferase; HbA1c, glycosylated haemoglobin; NIHSS, National Institute of Health Stroke Scale; FLI, Fatty Liver Index; FIB-4, Fibrosis-4.

**Table 2:** Prevalence of stroke risk factors, prevalence of patients with poor prognosis (Rankin 3-6) and mortality (Rankin 6) and prevalence of FIB-4>2.67 according to age (years) at the time of the first acute ischemic stroke.

	<30y (n=4)	30-39y (n=28)	40-49y (n=61)	50-59y (n=133)	60-69y (n=259)	70-79y (n=486)	80-89y (n=513)	>90y (n=117)	All ages (n=1601)
**Hypertension, n (%)**	0 (0)	7 (25.0)	32 (52.5)	89 (66.9)	176 (68.0)	399 (82.1)	414 (80.7)	94 (80.3)	1211 (75.7)
**Dyslipidemia, n (%)**	0 (0)	7 (25.0)	29 (47.5)	83 (62.4)	143 (55.2)	267 (54.9)	228 (44.4)	42 (35.9)	799 (49.9)
**Type 2 diabetes, n (%)**	0 (0)	2 (7.1)	13 (21.3)	41 (30.8)	94 (36.3)	200 (41.2)	159 (31.0)	26 (22.2)	535 (33.4)
**Obesity (BMI >30kg/m2), n (%)**	0 (0)	7 (25.0)	21 (24.4)	38 (28.6)	95 (36.7)	129 (26.5)	107 (20.9)	24 (20.5)	421 (26.3)
**Atrial fibrillation, n (%)**	1 (25.0)	2 (7.1)	2 (3.3)	9 (6.8)	61 (23.6)	182 (37.5)	249 (48.5)	71 (60.7)	577 (36.0)
**Rankin 3 – 6, n (%)**	0 (0)	3 (10.7)	9 (13.1)	22 (16.5)	66 (25.5)	198 (40.7)	296 (57.7)	96 (82.1)	689 (43.0)
**Mortality (Rankin 6), n (%)**	0 (0)	0 (0)	1 (1.6)	3 (2.3)	9 (3.5)	39 (8.0)	78 (15.2)	38 (32.5)	168 (10.5)
**FIB-4>2.67, n (%)**	0 (0)	0 (0)	0 (0)	0 (0)	7 (2.7)	69 (27.0)	129 (50.4)	51 (19.9)	256 (16.0)

### Prevalence of NAFLD and liver fibrosis

**Figure 2:**
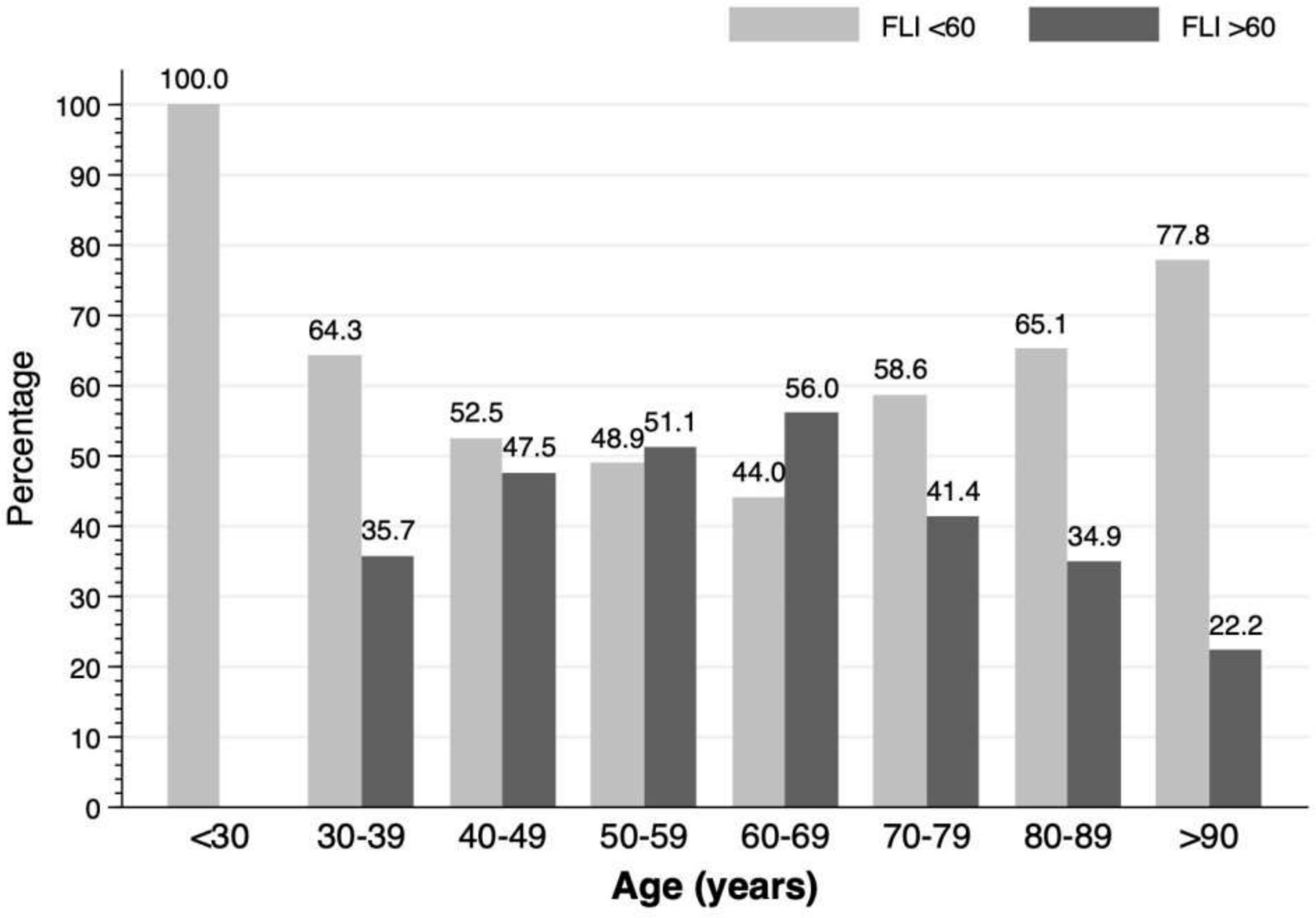
Prevalence of non-alcoholic fatty liver disease according to age decade in patients with a first acute ischemic stroke.

The proportion of patients with a FLI score >60, between 30-60 and <30 was 41.1%, 31.2% and 27.7%, respectively. The distribution of NAFLD according to age is shown in **Figure 2**. Differences in prevalence were observed in the different decades: in 30-39 years: 35.7%; in 40-49: 47.5%; in 50-59: 51.1%, in 60-69: 56%, in 70-79: 41.4%; in 80-89: 34.9% (p<0.001). No patient under 30 years of age had FLI >60.

**Figure 3:**
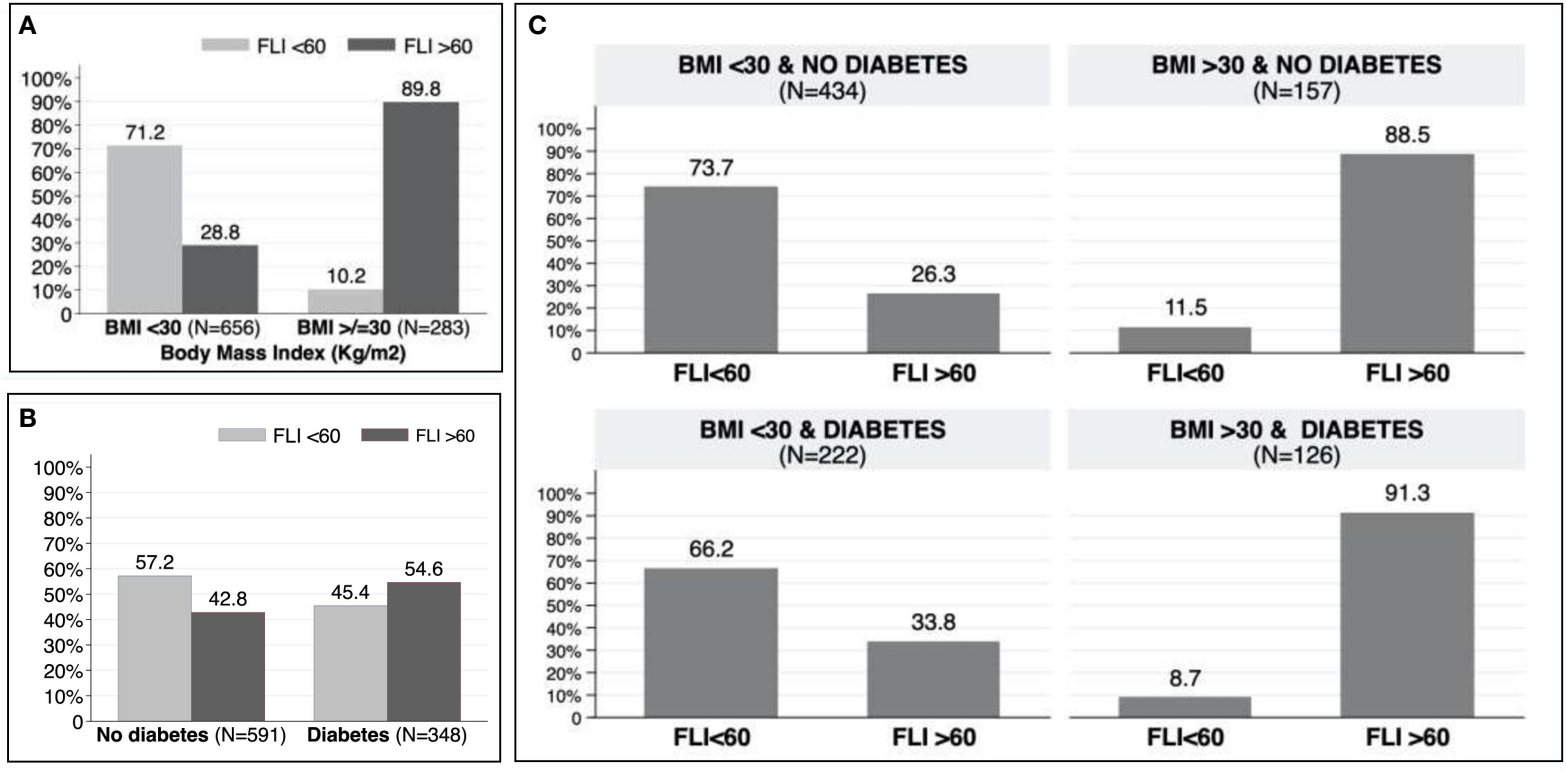
**(A)** Prevalence of non-alcoholic fatty liver disease in patients with and without obesity. **(B)** Prevalence of non-alcoholic fatty liver disease in patients with and without type 2 diabetes mellitus. **(C)** Prevalence of non-alcoholic fatty liver disease according to obesity and/or type 2 diabetes mellitus.

In patients aged between 40 and 79 years old, the prevalence of NAFLD was 47.2%. Patients with NAFLD were younger (68 vs. 71; p=0.002), had a higher BMI (Kg/m^2^) (30.8 vs. 25.0; p<0.001), and a higher prevalence of hypertension (80.1% vs. 68.8%; p<0.001), T2DM (42.9% vs. 31.9%; p<0.001) and dyslipidemia (61.9% vs. 50.0%; P<0.001). There were no differences on the presence of AF between both groups (27.5% vs. 26.6%; p=0.750). **Table 3** shows the differences between patients with FLI>60 and FLI<60 in patients aged 40-79 years old. Similar results were found in the whole cohort (**Supplementary Table 1**). According to risk factors, the proportion of patients with FLI >60 increased to 54.6% in patients with T2DM (**Figure 3A**), to 89.8% in patients with obesity (**Figure 3B**), and up to 91.3% in patients with both T2DM and obesity (**Figure 3C**). Even without T2DM or obesity, the 26.3% of patients showed a FLI>60 (**Figure 3C**).

**Table 3:** Baseline characteristics of patients between 40 and 79 years old with a first acute ischemic stroke (n=939) and according to the presence or absence of non-alcoholic fatty liver evaluated with Fatty Liver Index.

	N = 939	FLI <60 (N = 496)	FLI ≥60 (N = 443)	p-Value
SOCIODEMOGRAPHIC DATA AND COMORBIDITIES				
** Age** (years)	70 (61 – 76)	71 (62 – 77)	68 (61 – 76)	0.002
** Female**, n (%)	401 (42.7)	201 (40.5)	200 (45.2)	0.153
** Caucasian ethnicity**, n(%) (n=925)	857 (92.7)	456 (93.1)	401 (92.2)	0.610
** Tobacco habit**, n (%) (n=921)				
** **Never smoker	503 (54.6)	246 (50.9)	257 (58.7)	0.034
** **Ex-smoker > 5 years	129 (14.0)	68 (14.1)	61 (13.9)	
Active smoker or ex-smoker < 5 years	289 (31.4)	169 (35.0)	120 (27.4)	
** Abdominal circumference** (cm)	100 (90 – 110)	91 (84 – 98)	110 (102 – 117)	<0.001
** BMI** (kg/m2)	27.3 (24.4 – 30.8)	25.0 (22.9 – 27.0)	30.8 (28.1 – 33.9)	<0.001
** **BMI >30kg/m^2^, n (%)	283 (30.1)	29 (5.9)	254 (57.3)	<0.001
** High blood pressure**, n (%)	696 (74.1)	341 (68.8)	355 (80.1)	<0.001
** Type 2 Diabetes**, n (%)	348 (37.1)	158 (31.9)	190 (42.9)	<0.001
** Dyslipidemia**, n (%)	522 (55.6)	248 (50.0)	274 (61.9)	<0.001
** Atrial fibrillation**, n (%)	254 (27.1)	132 (26.6)	122 (27.5)	0.750
BLOOD TEST				
** AST** (U/L)	18 (15 – 32)	17 (15 – 22)	18 (15 – 25)	0.025
** ALT** (U/L)	16 (12 – 23)	15 (12 – 21)	18 (14 – 26)	<0.001
** GGT** (U/L)	24 (16 – 39)	20 (14 – 31)	32 (21 – 50)	<0.001
** Albumin** (g/dl) (n=731)	3.8 (3.6 – 4.1)	3.8 (3.6 – 4.1)	3.9 (3.6 – 4.1)	0.046
** Platelets** (·10^9^/L)	222.0 (186.0 – 267.0)	222.0 (186.5 – 269.0)	222.0 (185.0 – 264.0)	0.582
** Total cholesterol** (mg/dl) (n=920)	176 (145 – 208)	171 (139 – 199)	184 (154 – 213)	<0.001
** HDL** (mg/dl) (n=893)	44 (36 – 54)	45 (37 – 56)	43 (35 – 51)	<0.001
** Triglycerides** (mg/dl)	119 (87 – 162)	103 (77 – 136)	141 (107 – 188)	<0.001
** HbA1c** (%) (n=803)	5.8 (5.3 – 6.9)	5.7 (5.2 – 6.5)	6.0 (5.4 – 7.2)	<0.001
STROKE-RELATED VARIABLES				
** Stroke ethiology**, n (%)				
** **Large artery disease	141 (15.0)	76 (15.3)	65 (14.7)	0.848
** **Cardioembolic	257 (27.4)	132 (26.6)	125 (28.2)	
** **Small vessel disease	257 (27.4)	135 (27.2)	122 (27.5)	
** **Undetermined	180 (19.2)	93 (18.8)	87 (19.6)	
** **Others	104 (11.1)	60 (12.1)	44 (9.9)	
** NIHSS**, n (%) (n=933)				
** **<7	665 (71.3)	340 (69.0)	325 (73.9)	0.217
** **7 – 14	139 (14.9)	77 (15.6)	62 (14.1)	
** **>14	129 (13.8)	76 (15.4)	53 (12.1)	
** Rankin**, n (%)				
** **0 – 2	645 (68.7)	335 (67.5)	310 (70.0)	0.173
** **3 – 5	242 (25.8)	127 (25.6)	115 (26.0)	
** **6	52 (5.5)	34 (6.9)	18 (4.0)	
** Death,** n(%)	52 (5.5)	34 (6.9)	18 (4.0)	0.062

BMI, body mass index; AST, aspartate aminotransferase; ALT, alanine aminotransferase; GGT, gamma glutamyl transferase; HbA1c, glycosylated haemoglobin; NIHSS, National Instituted of Health Stroke Scale; FLI, Fatty Liver Index; FIB-4, Fibrosis-4.

**Figure 4:**
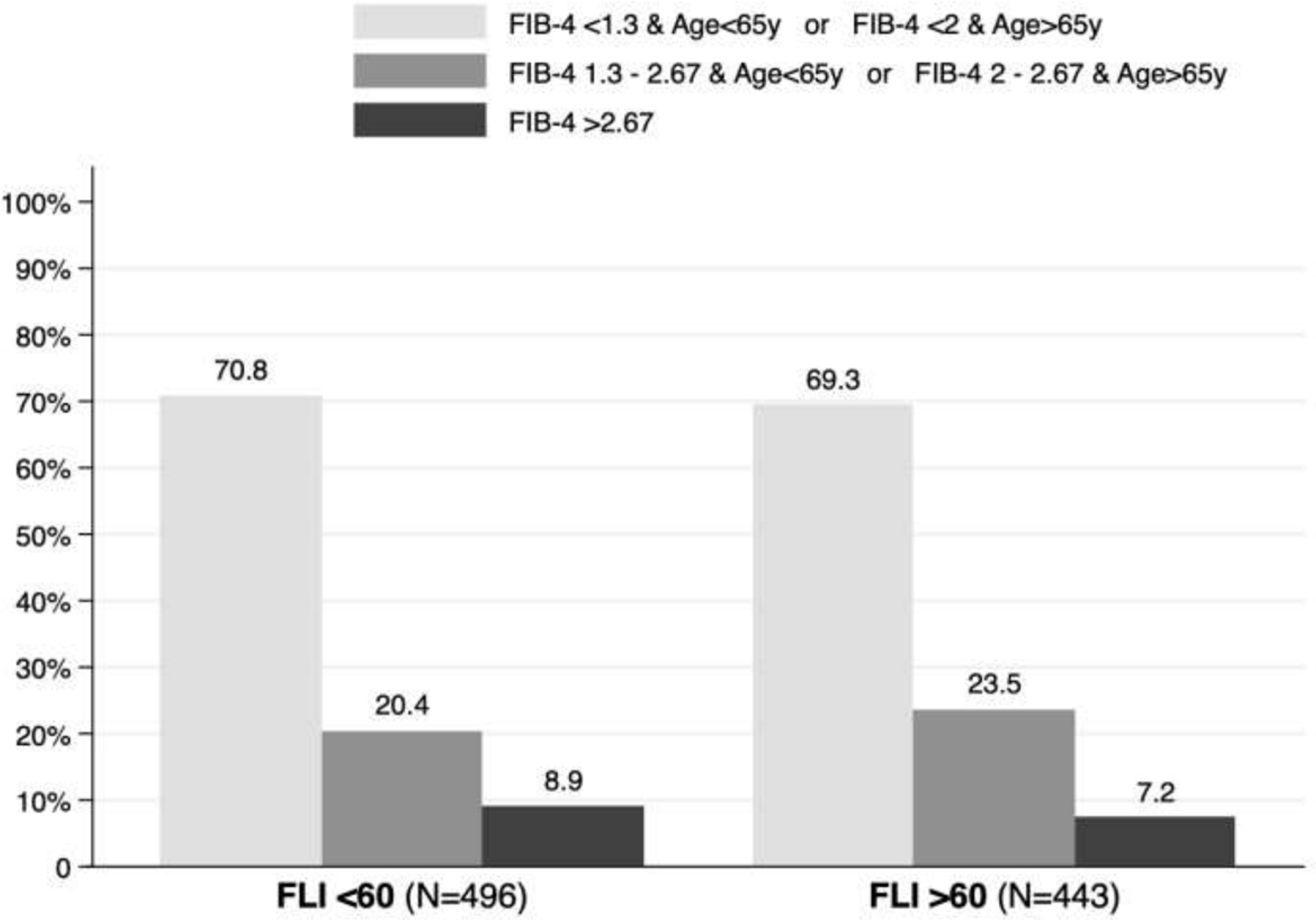
Fibrosis-4 (FIB-4) adjusted by age in patients with and without non-alcoholic fatty liver disease.

Advanced liver fibrosis was excluded in 70.8% of patients with a FLI<60 and in 69.3% of patients with a FLI>60 using FIB-4 adjusted for age. In contrast, a FIB-4 >2.67, was present in 8.9% and 7.2% of patients, respectively (**Figure 4**).

### Severity and prognosis of AIS

**Figure 5:**
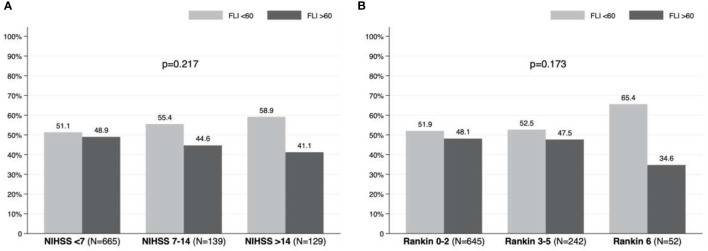
Prevalence of non-alcoholic fatty liver disease according to: **(A)** severity (NIHSS) and **(B)** prognosis (modified Rankin Scale) in patients with acute ischemic stroke.

Stroke severity was mild in 665 patients (71.3%), moderate in 139 (14.9%), and severe in 129 (13.8%). In terms of prognosis, 242 (25.8%) patients presented moderate-severe disability (mRS 3 – 5) at three months, and 52 (5.5%) died. **Table 2** details the prevalence of poor prognosis (mRS 3 – 6) according to age at the stroke. The prevalence of poor outcome and mortality was 31.3% and 5.5% of patients aged between 40 and 79 years, while poor outcome and mortality was found in 62.2% and 18.4% of patients over 80 years old. **Table 3** and **Supplementary Table 1** show the severity and prognosis of the AIS according to FLI. The proportion of patients with FLI >60 in each of the NIHSS and mRS groups is depicted graphically in **Figure 5**; note that no differences on severity and disability of the AIS were found based on the presence or absence of NAFLD (p=0.217 for NIHSS and p=0.173 for mRS).

We evaluated the implication of age, gender, smoking habit, comorbidities, FLI and FIB-4, as well as the individual variables used to calculate these scores, in stroke prognosis (**Table 4**). We performed two models of multivariate analyses, including those variables that were significant with p<0.05 in univariate analysis. On the first model FIB-4 was included as the percentage of patients with FIB-4>2.67 and the second model included the variables for FIB-4 calculation separately (**Table 5**). We found that female gender (aOR= 1.74, 95%CI [1.24-2.44], p=0.001), T2DM (aOR= 2.05, 95%CI [1.51-2.79]; p<0.001), AF (aOR= 1.64, 95%CI [1.18-2.28], p=0.003) and FIB-4 >2.67 (aOR= 2.82, 95%CI [1.68-4.73], p<0.001) were independent risk factors of poorer outcomes. On the second model, evaluating the variables that constitute the FIB-4 separately, age was the only variable of FIB-4 that was related with worse results. None of the variables of liver function test nor platelets were associated with outcomes (**Table 5**).

**Table 4:** Univariate analysis to assess factors related to worse prognosis (Rankin 0-3 vs. Rankin 3-6) in patients between 40 and 79 years old with a first acute ischemic stroke (n=939).

Rankin 0-2 (N=645)	Rankin 3-6 (N = 294)	p-Value
SOCIODEMOGRAPHIC DATA AND COMORBIDITIES			
**Age** (years)	66 (59 – 74)	74 (67 – 77)	<0.001
**Female**, n (%)	246 (38.1)	155 (52.7)	<0.001
**Tobacco habit**, n (%) (n=921)			
Never smoker	325 (51.2)	178 (62.2)	0.002
Ex-smoker > 5 years	88 (13.9)	41 (14.3)	
**Active smoker or ex-smoker < 5 years**	222 (34.9)	67 (23.4)	
**High blood pressure**, n (%)	457 (70.9)	239 (81.3)	0.001
**Type 2 Diabetes**, n (%)	209 (32.4)	139 (47.3)	<0.001
**Dyslipidemia**, n (%) (n=653)	357 (55.4)	165 (56.1)	0.825
**Atrial fibrillation**, n (%)	144 (22.3)	110 (37.4)	<0.001
FFATTY LIVER INDEX (FLI)			
**FLI**	57 (32 – 80)	57 (32 – 81)	0.999
**FLI >60**, n (%)	310 (48.1)	133 (45.2)	0.421
**Abdominal circumference** (cm)	100 (90 – 109)	100 (90 – 110)	0.549
**BMI** (kg/m2)	27.2 (24.5 – 30.7)	27.5 (24.2 – 31.2)	0.666
BMI >30kg/m^2^, n (%)	188 (29.2)	95 (32.3)	0.327
**GGT** (U/L)	24 (16 – 39)	24 (16 – 41)	0.966
**Triglycerides** (mg/dl)	123 (90 – 166)	111 (83 – 150)	0.001
Triglycerides >150 mg/dl	214 (33.2)	75 (25.5)	0.018
FIBROSIS-4 (FIB-4)			
**FIB-4**	1.31 (0.95 – 1.74)	1.57 (1.13 – 2.13)	<0.001
**FIB-4** >2.67, n (%)	30 (4.7)	46 (15.7)	<0.001
**AST** (U/L)	19 (14 – 23)	18 (15 – 25)	0.153
AST >40U/L	27 (4.2)	26 (8.8)	0.004
**ALT** (U/L)	17 (13 – 24)	15 (12 – 21)	0.009
ALT >40U/L	45 (7.0)	18 (6.1)	0.627
**Platelets** (·10^9^/L)	222.0 (186.0 – 268.0)	222.0 (185.0 – 266.0)	0.936

FLI, Fatty Liver Index; BMI, body mass index; GGT, gamma glutamyl transferase; FIB-4, Fibrosis-4; AST, aspartate aminotransferase; ALT, alanine aminotransferase. Univariate analysis performed with Chi-Square for categorical variables and U Mann-Whitney tests.

**Table 5:** Multivariate analysis to assess factors related to worse prognosis (Rankin 0-3 vs. Rankin 3-6) in patients between 40 and 79 years old with a first acute ischemic stroke (n=939).

	Model 1		Model 2	
	OR (IC95%)	p-Value	OR (IC95%)	
SOCIODEMOGRAPHIC DATA AND COMORBIDITIES				
**Age** (years)			1.05 (1.03 – 1.07)	<0.001
**Female**, n (%)	1.74 (1.24 – 2.44)	0.001	1.69 (1.20 – 2.38)	0.003
**Tobacco habit**, n (%) (n=921)				
Never smoker	Ref.		Ref.	
Ex-smoker > 5 years	1.16 (0.72 – 1.85)	0.548	1.15 (0.71 – 1.85)	0.564
**Active smoker or ex-smoker < 5 years**	0.92 (0.63 – 1.34)	0.663	1.20 (0.80 – 1.80)	0.369
**High blood pressure**, n (%)	1.43 (0.99 – 2.06)	0.053	1.32 (0.91 – 1.90)	0.144
**Type 2 Diabetes**, n (%)	2.05 (1.51 – 2.79)	<0.001	1.88 (1.38 – 2.56)	<0.001
**Dyslipidemia**, n (%) (n=653)				
**Atrial fibrillation**, n (%)	1.64 (1.18 – 2.28)	0.003	1.43 (1.03 – 2.01)	0.034
FATTY LIVER INDEX (FLI)				
**FLI**				
**FLI >60**, n (%)				
**Abdominal circumference** (cm)				
**BMI** (kg/m2)				
BMI >30kg/m^2^, n (%)				
**GGT** (U/L)				
**Triglycerides** (mg/dl)				
Triglycerides >150 mg/dl	0.73 (0.52 – 1.02)	0.065	0.78 (0.55 – 1.10)	0.155
FIBROSIS-4 (FIB-4)				
**FIB-4**				
**FIB-4 >2.67, n (%)**	2.82 (1.68 – 4.73)	<0.001		
**AST** (U/L)				
AST >40U/L			1.80 (0.97 – 3.36)	0.064
**ALT** (U/L)				
ALT >40U/L				
**Platelets** (·10^9^/L)				

FLI, Fatty Liver Index; BMI, body mass index; GGT, gamma glutamyl transferase; FIB-4, Fibrosis-4; AST, aspartate aminotransferase; ALT, alanine aminotransferase. Two models of multivariate analysis performed binary logistic regression.

## Discussion

NAFLD is the most common chronic liver disease in adult population and its prevalence is expected to increase (1, 35). Although NAFLD has been related with cardiovascular events, most studies are focused on cardiological events and less has been published on cerebrovascular events. In this study, with a large cohort of 1601 patients admitted for first AIS, we provide data on the prevalence of NAFLD, and its impact on the severity and the prognosis of AIS. In our study, NAFLD affected 41% of patients. This prevalence was much higher than described in the adult population in our area (3). Remarkably, the prevalence of NAFLD was higher at intermediate ages with prevalence above 50% in patients aged between 50 and 70 years and then decreased in subsequent decades. We found that patients with NAFLD, were significantly younger compared to patients without NAFLD (78 vs. 74 years). Minor or non-significant differences were reported in previous cohorts (36, 37). This high prevalence of NAFLD in our cohort, could be explained because it is a selected cohort of patients, with a high prevalence of risk factors (T2DM, hypertension, dyslipidemia) that both AIS and NAFLD share.

Given the high prevalence of metabolic dysfunction and NAFLD in adult population (1–3), and the increasing of its frequency expected in the near future (35), a higher frequency of hepatic and extrahepatic NAFLD-related events is expected. Therefore, multidisciplinary management of diseases related to metabolic dysfunction is mandatory. Moreover, NAFLD is a progressive disease with a long asymptomatic phase that allows an opportunity for early diagnosis and prevention of progression. Hence, in recent years it has been suggested that screening of NAFLD in early stages should be advisable (38). It has been shown that screening of liver fibrosis is cost-effective in general population and even more in high risk groups (39). Therefore, defining high risk patients for chronic liver disease is an important issue to better select screening strategies. The results of our study showed that patients with a first episode of AIS had a high prevalence of NAFLD and should be considered as high-risk patients, and therefore tributary of an active evaluation of liver disease, especially those at intermediate ages.

The risk factors of AIS have been previously described. Based on data from the Spanish EPICES stroke registry, the authors reported a prevalence of hypertension, dyslipidemia, T2DM, and obesity of 67%, 36%, 31%, and 14% respectively (40). We found higher prevalence of these risk factors in our cohort, and even higher in patients with NAFLD, especially in younger patients. Another important point is that NAFLD has been proposed as a risk factor of AF, and the association seemed to be greater as liver disease progresses (esteatohepatitis, liver fibrosis, cirrhosis). In addition, it was suggested that advancing age and greater comorbidity, in particular T2DM, increase the risk of AF among the NAFLD subjects (41). In our cohort, AF was present in one third of patients, and no difference was observed between patients with and without NAFLD. As expected, AF was most prevalent in elderly patients, being present in 51% of patients older than 80 years old. Consequently, the different distribution of NAFLD and comorbidities in the age groups, suggest that younger patients had a stronger metabolic background while in patients experiencing a stroke at older ages (>80 years), other mechanisms are involved in the occurrence of the stroke such as atrial fibrillation.

As previously reported (24, 42), in our cohort we have confirmed that the risk of stroke and its morbidity and mortality increases with age. For this reason, in the present study we focused the analysis on the impact of NAFLD on stroke outcomes in patients between 40 and 79 years. In those patients the prevalence of NAFLD was 47%. Most of AIS in our cohort were mild and had good prognosis. We did not find differences in the initial severity of the stroke evaluated by NIHSS or in the disability and mortality at 3 months between patients with or without NAFLD. In contrast to these results, two studies found that patients with NAFLD had more severe AIS (36, 37), and only Abdeldyem et al. related NAFLD with a worse prognosis of AIS. However, it should be considered that both studies had a lower number of patients than our cohort, and importantly, NAFLD was defined based on the presence of transaminase abnormalities in the absence of other liver diseases. It is important to note that it has been described that aspartate-aminotransferase can increase during stroke related to stroke volume (43). Therefore, evaluation of NAFLD based on levels of transaminases in the setting of an ischemic event, may not be accurate enough.

Further, liver fibrosis was evaluated in our cohort using the FIB-4, the most validated serologic marker. Advanced liver fibrosis was excluded in more than 70% of patients using FIB-4 adjusted for age, and <10% had high probability of advanced fibrosis. However, due to the advanced age of our cohort, we consider that the use of FIB-4 could overestimate the risk of liver fibrosis in our study. Therefore, other non-invasive methods such as transient elastography could be more accurate (44). When evaluating the factors that predict poor prognosis after a first episode of stroke, we found that female gender, T2DM and atrial fibrillation are related to worse outcomes. Although it seems that the FIB-4 >2.67 can be independently related to a worse prognosis of AIS, when evaluating the variables that constitute the FIB-4 separately, age is the only variable related to worse results.

The present study has some limitations. First, its retrospective design carries its inherent bias, but it was based on the analysis of a well characterized prospective cohort of consecutive patients with a large sample size that include anthropometric variables. Second, the diagnosis of NAFLD and liver fibrosis has been made based on non-invasive serological tests and therefore we do not have another method such ultrasound, controlled attenuation parameter and transient elastography of FibroScan®, or histology to compare. However, even if FLI is not the gold standard for NAFLD evaluation, it is an easy score to apply in clinical practice that has been associated to hepatic-related death, cardiovascular disease mortality (45) and incident cardiovascular events (46). Moreover, given the relation of aspartate-aminotransferase elevations in patients with a stroke (43) we consider an advantage using FLI in those patients as it not consider transaminases. Third, the presence and characteristics of carotid plaques were not evaluated, neither the carotid artery intima-media thickness, that have been associated to atherosclerosis and may be influenced by NAFLD (47).

In conclusion, presence of NAFLD did not impact on disability and death after the stroke. However, NAFLD is highly prevalent in patients with a first episode of ischemic stroke, especially at intermediate ages. Hence, those patients should be considered as high-risk patients and should be screened. Moreover, metabolic risk factors and NAFLD are more prevalent in patients with an ischemic stroke at younger ages while other factors such as atrial fibrillation are more frequent in the elderly.

## Data availability statement

The raw data supporting the conclusions of this article will be made available by the authors, if asked.

## Ethics statement

The studies involving human participants were reviewed and approved by Comitè Ètic d’Investigació Clínica - Parc de Salut Mar”, study reference (2008/3083/I). The patients/participants provided their written informed consent to participate in this study.

## Author contributions

Study concept and design: TB. Acquisition of data and technical support: LC, AS-V, AR-C, EG-S, and EC-G. Analysis and interpretation of data: LC, AS-V, TB. Draft of the manuscript: LC, AS-V, and TB. Critical revision of the manuscript for important intellectual content: AR-C, EG-S, and EC-G. Statistical analysis: LC, AS-V, and TB. Study supervision: TB. All authors contributed to the article and approved the submitted version.
