# Supplementary material for: High prevalence of non-alcoholic fatty liver disease in patients with a first episode of acute ischemic stroke. Impact on disability and death
Source: Front Endocrinol (Lausanne). 2022 Dec 16;13:1003878. doi: 10.3389/fendo.2022.1003878 (PMC9800794; doi:10.3389/fendo.2022.1003878)
Supplement: Supplementary file 1 [file Table_1.docx]

**SUPPLEMENTARY TABLE**

**Supplementary table 1: Baseline characteristics of patients with a first acute ischemic stroke (n=1601) and according with the presence or absence of non-alcoholic fatty liver evaluated with non-invasive Fatty Liver Index.**BMI: body mass index. AST: aspartate aminotransferase. ALT: alanine aminotransferase. GGT: gamma glutamyl transferase. HbA1c: glycosylated haemoglobin. NIHSS: National Instituted of Health Stroke Scale. FLI: Fatty Liver Index. FIB-4: Fibrosis-4.

|  | **ALL PATIENTS**  **(N= 1601)** | **FLI <60**  **(N=943)** | **FLI ≥60**  **(N=658)** | p |
| --- | --- | --- | --- | --- |
| **SOCIODEMOGRAPHIC DATA AND COMORBIDITIES** | | | | |
| **Age** (years) | 77 (66 – 83) | 78 (69 – 84) | 74 (63 – 81) | <0.001 |
| **Female**, n (%) | 839 (52.4) | 498 (52.8) | 341 (51.8) | 0.697 |
| **Caucasian ethnicity**, n(%) (n=1575) | 1491 (94.7) | 887 (95.4) | 604 (93.6) | 0.132 |
| **Tobacco habit**, n (%) (n=1576) |  |  |  |  |
| Never smoker | 1043 (66.2) | 605 (65.6) | 438 (67.1) | 0.618 |
| Ex-smoker > 5 years | 198 (12.6) | 114 (12.4) | 84 (12.9) |  |
| Active smoker or ex-smoker < 5 years | 335 (21.3) | 204 (22.1) | 131 (20.1) |  |
| **Abdominal circumference** (cm) | 98 (90 – 108) | 90 (83 – 98) | 110 (102 – 118) | <0.001 |
| **BMI** (kg/m2) | 26.8 (24.2 – 30.2) | 24.8 (22.8 – 27.0) | 30.5 (27.8 – 33.6) | <0.001 |
| BMI <25 kg/m^2^, n (%) | 535 (33.4) | 495 (52.5) | 40 (6.1) | <0.001 |
| BMI 25 - 30 kg/m^2^, n (%) | 645 (40.3) | 397 (42.1) | 248 (37.7) |  |
| BMI >30kg/m^2^, n (%) | 421 (26.3) | 51 (5.4) | 370 (56.2) |  |
| **High blood pressure**, n (%) | 1211 (75.6) | 675 (71.6) | 536 (81.5) | <0.001 |
| **Type 2 Diabetes**, n (%) | 535 (33.4) | 265 (28.2) | 270 (41.0) | <0.001 |
| **Dyslipidemia**, n (%) | 799 (49.9) | 429 (45.5) | 370 (56.2) | <0.001 |
| **Atrial fibrillation**, n (%) | 577 (36.0) | 349 (37.0) | 228 (34.7) | 0.333 |
| **BLOOD TEST** |  |  |  |  |
| **AST** (U/L) | 18 (15 – 24) | 18 (15 – 23) | 19 (15 – 26) | 0.008 |
| **ALT** (U/L) | 15 (12 – 21) | 14 (11 – 20) | 18 (13 – 26) | <0.001 |
| **GGT** (U/L) | 23 (15 – 38) | 19 (13 – 30) | 33 (20 – 56) | <0.001 |
| **Albumin** (g/dl) (n=1217) | 3.8 (3.5 – 4.0) | 3.8 (3.5 – 4.0) | 3.8 (3.6 – 4.1) | <0.001 |
| **Platelets** (·10^9^/L) | 219.0 (183.0 – 265.0) | 215.0 (178.0 – 261.0) | 219.0 (181.0 – 270.0) | 0.397 |
| **Total cholesterol** (mg/dl) (n=1562) | 172 (142 – 200) | 166 (138 – 194) | 176 (147 – 207) | <0.001 |
| **HDL** (mg/dl) | 46 (38 – 56) | 47 (39 – 58) | 44 (36 – 53) | <0.001 |
| **Triglycerides** (mg/dl) | 111 (84  – 147) | 97 (74 – 128) | 131 (102 – 176) | <0.001 |
| **HbA1c** (%) (n=1347) | 5.8 (5.3 – 6.6) | 5.6 (5.2 – 6.3) | 6 (5.3 – 6.6) | <0.001 |
| **STROKE-RELATED VARIABLES** |  |  |  |  |
| **Stroke ethiology**, n (%) |  |  |  |  |
| Atherothrombotic | 190 (11.9) | 107 (11.4) | 83 (12.6) | 0.331 |
| Cardioembolic | 565 (35.3) | 196 (20.8) | 229 (34.8) |  |
| Lacunar | 355 (22.2) | 336 (35.6) | 159 (24.2) |  |
| Indeterminate complete | 299 (18.7) | 183 (19.4) | 116 (17.6) |  |
| Others | 192 (12.0) | 121 (12.8) | 71 (10.8) |  |
| **NIHSS**, n (%) (n=1592) |  |  |  |  |
| <7 | 1023 (64.3) | 574 (61.1) | 449 (68.8) | 0.007 |
| 7 – 14 | 309 (19.4) | 195 (20.8) | 114 (17.5) |  |
| >14 | 260 (16.3) | 170 (18.1) | 90 (13.8) |  |
| **Rankin**, n (%) |  |  |  |  |
| 0 – 2 | 912 (57.0) | 515 (54.6) | 397 (60.3) | 0.012 |
| 3 – 5 | 521 (32.5) | 313 (33.2) | 208 (31.6) |  |
| 6 | 168 (10.5) | 115 (12.2) | 53 (8.1) |  |
| **Death,** n(%) | 168 (10.5) | 115 (12.2) | 53 (8.1) | 0.008 |
